# Fish Oil Supplementation Improves the Repeated-Bout Effect and Redox Balance in 20–30-Year-Old Men Submitted to Strength Training

**DOI:** 10.3390/nu15071708

**Published:** 2023-03-31

**Authors:** Gustavo Barquilha, Cesar Miguel Momesso Dos Santos, Kim Guimaraes Caçula, Vinícius Coneglian Santos, Tatiana Geraldo Polotow, Cristina Vardaris Vasconcellos, José Alberto Fernandes Gomes-Santos, Luiz Eduardo Rodrigues, Rafael Herling Lambertucci, Tamires Duarte Afonso Serdan, Adriana Cristina Levada-Pires, Elaine Hatanaka, Maria Fernanda Cury-Boaventura, Paulo Barbosa de Freitas, Tania Cristina Pithon-Curi, Laureane Nunes Masi, Marcelo Paes Barros, Rui Curi, Renata Gorjão, Sandro Massao Hirabara

**Affiliations:** 1 Interdisciplinary Post-Graduate Program in Health Sciences, Institute of Physical Activity Sciences and Sports, Cruzeiro do Sul University, Sao Paulo 01506-000, Brazil; 2 ENAU Faculty, Ribeirão Pires 09424-130, Brazil; 3 United Metropolitan Colleges, Centro Universitário FMU, Sao Paulo 01503-001, Brazil; 4 Department of Physiology and Biophysics, Institute of Biomedical Sciences, University of Sao Paulo, Sao Paulo 05508-000, Brazil; 5 Institute of Health and Society, Federal University of Sao Paulo, Santos 11015-020, Brazil; 6 Department of Molecular Pathobiology, New York University, New York, NY 10010, USA; 7 Instituto Butantan, Sao Paulo 05503-900, Brazil

**Keywords:** non-linear strength training, *n-3* polyunsaturated fatty acids, inflammation, muscle damage, oxidative stress

## Abstract

Herein, we investigated the effect of fish oil supplementation combined with a strength-training protocol, for 6 weeks, on muscle damage induced by a single bout of strength exercise in untrained young men. Sixteen men were divided into two groups, supplemented or not with fish oil, and they were evaluated at the pre-training period and post-training period. We investigated changes before and 0, 24, and 48 h after a single hypertrophic exercise session. Creatine kinase (CK) and lactate dehydrogenase (LDH) activities, plasma interleukin-6 (IL-6) and C-reactive protein (CRP) levels, and the redox imbalance were increased in response to the single-bout session of hypertrophic exercises at baseline (pre-training period) and decreased during the post-training period in the control group due to the repeated-bout effect (RBE). The fish oil supplementation exacerbated this reduction and improved the redox state. In summary, our findings demonstrate that, in untrained young men submitted to a strength-training protocol, fish oil supplementation is ideal for alleviating the muscle injury, inflammation, and redox imbalance induced by a single session of intense strength exercises, highlighting this supplementation as a beneficial strategy for young men that intend to engage in strength-training programs.

## 1. Introduction

Muscle damage induced by unusual eccentric exercises results in several skeletal muscle changes, including the release of muscle enzymes into the blood, a reduction in muscle strength, an increase in muscle soreness, and the activation of the inflammatory process and oxidative stress [1]. Nowadays, it is known that these alterations, when well-controlled, are required for adequate and complete muscle recovery [1]. However, when the inflammatory response and oxidative stress are exacerbated, an imbalance occurs in these processes, impairing or delaying muscle repair and regeneration [1,2]. This condition also causes high ATP generation via anaerobic metabolism, muscle inflammation, and oxidative stress. Oxidative stress leads to a change in iron homeostasis and antioxidant depletion, as observed by variations in the reduced: oxidized glutathione ratio [3,4]. Indirect markers of muscle damage—e.g., the plasma activities of creatine kinase (CK) and lactate dehydrogenase (LDH) [4,5,6]—are frequently evaluated to monitor the efficiency and risks of strength-training protocols in exercising subjects and athletes. Usually, the plasma activities of these enzymes increase within 6 to 8 h after a strength exercise session, peaking between 48 and 72 h and remaining elevated for up to 7 days [5,7]. Delayed-onset muscle soreness associated with muscle injury also peaks between 24 and 48 h post-exercise, and it is more pronounced in non-trained individuals and older people than in high-performance strength athletes [5,7].

After a muscle injury induced by eccentric contractions, the inflammatory response initiates tissue repair and regeneration [1,8]. This response involves the release of cytokines, including interleukin-6 (IL-6), interleukin-1b (IL-1b), and tumor necrosis factor-a (TNF-α). IL-6 is the main cytokine to increase after physical exercise [9,10]. These cytokines also increase in strenuous, high-intensity, and intermittent exercises [11,12]. These three pro-inflammatory cytokines act on the liver, stimulating the production and release of C-reactive protein (CRP), an indicator of systemic acute inflammation [13]. After a single extenuating aerobic or strength exercise session, CRP plasma levels increase [14,15].

The term the “repeated-bout effect” (RBE) commonly refers to the protective adaptation against muscle injury caused by an identical or a similar bout of eccentric exercises after a single bout of eccentric exercise or after a period of strength training [16,17,18]. This phenomenon has been observed in several animal and human models and usually lasts from weeks to months [19]. The mechanisms involved in the RBE are not entirely understood, but several theories have been proposed, including mechanical, cellular, and neural adaptations [18,19]. Potential interventions for increasing this effect can also help to decrease the impact of muscle damage in subjects during their training program.

Muscle disorders (e.g., lesions, oxidative stress, inflammation, and atrophy) often occur in several conditions, including in exercise-induced injuries and chronic diseases (e.g., obesity, diabetes, metabolic syndrome, and cardiovascular diseases). Cryotherapy [20,21,22] and the administration of antioxidant and anti-inflammatory agents [23,24], including omega-3 polyunsaturated fatty acids (*n-3* PUFAs) [25,26,27,28], have been proposed to provide a protective effect in these muscle disorders or exercise-induced muscle injury. The main anti-inflammatory *n-3* PUFAs comprise eicosapentaenoic acid (EPA) and docosahexaenoic acid (DHA), which have been demonstrated to reduce plasma lipids [3,29,30], oxidative stress conditions [31,32], insulin sensitivity [31,33,34], and inflammation [33,35]. Similar to regular and moderate aerobic exercise, *n-3* PUFAs reduce fat mass [36,37] and cardiovascular risks [38,39], but they do not raise physical capabilities [40] or performance [41,42]. Interestingly, when young endurance athletes are supplemented with highly purified *n-3* PUFAs (2.1 g DHA + 240 mg EPA per day for 10 wks), they present reduced muscle damage (plasma CK and LDH activities), inflammatory markers (plasma IL-6 and IL-1β), and muscle soreness after an eccentric-induced muscle damage exercise session [43].

It was observed that *n-3* PUFAs increase muscle strength gain in older women submitted to a strength-training program [44] or resistance exercise training [44,45,46]. Due to their anti-inflammatory effects, *n-3* PUFAs have beneficial effects in some diseases, such as neurodevelopmental disorders related to oxidative stress (Rett Syndrome) [47], multiple sclerosis [48], and depression [49]. The effects of fish oil supplementation on muscle damage induced by different physical exercise protocols have also been demonstrated in several studies. Acute supplementation (3 days, 3 g per day) with krill oil (a natural source of *n-3* PUFAs) was sufficient to reduce muscle damage induced by exercise (plasma CK activity) and malondialdehyde content, a stress oxidative marker, but it did not have a significant effect on inflammatory cytokines in resistance-trained young men [28]. In another study, it was observed that supplementation with fish oil for 6 wks in untrained young men induces a reduction in oxidative stress markers (thiobarbituric-acid-reactive substances and H_2_O_2_-induced DNA damage) after a single bout of eccentric exercise, but it did not have an effect on muscle damage markers or muscle soreness [50]. In highly trained athletes (power training or high-intensity interval training activities), krill oil supplementation (2.5 g per day), for 12 weeks, was associated with reduced oxidative stress after a high-intensity physical exercise session [51]. In untrained young men, fish oil supplementation (600 mg EPA and 260 mg DHA per day), for 8 weeks, was able to attenuate the muscle strength loss, range of motion, muscle soreness, and plasma IL-6 increase induced by a session of maximal voluntary eccentric contractions of the elbow flexors [52]. Some of these effects (the range of motion and serum CK activity) were also observed when subjects were supplemented for a shorter period (4 weeks) [53]. Previously, fish oil supplementation (3 g per day) for 4 weeks was also associated with reduced muscle soreness, an increase in plasma IL-6, and muscle peak power after downhill running at 65% VO_2max_ for 60 min [54]. In another study, it was observed that fish oil supplementation (6 g per day, for 7 weeks) improved muscle recovery and decreased muscle soreness after a damaging eccentric exercise session in recreationally active participants [55]. In summary, previous studies have evaluated the modulation of *n-3* PUFA supplementation on muscle injury induced by a single session of damaging exercises in untrained and trained participants, as well as in athletes. However, there are no studies addressing the combined effect of fish oil supplementation and strength exercise training on muscle damage in untrained participants. Thus, our study aimed to demonstrate the effect of *n-3* PUFA supplementation in combination with a strength-training protocol for 6 weeks on the muscle injury, inflammation, and redox balance induced by a single bout of intense strength exercises in untrained young men. For this purpose, we evaluated the plasma levels of cytokines and C-reactive protein, cortisol and testosterone, the activities of creatine kinase and lactate dehydrogenase, and redox state parameters (total iron, heme iron, reduced and oxidized glutathione, and Trolox equivalent antioxidant capacity—TEAC).

## 2. Material and Methods

### 2.1. Participants

All experimental procedures were carried out following the approval of the Ethical Committee for Research of the Cruzeiro do Sul University (Protocol Number: 0392009) and performed in compliance with the Helsinki Declaration. Initially, a total of 21 healthy men, between 20 and 30 years old, were eligible to participate in the study. All participants were classified as physically active using the International Questionnaire of Physical Activity, but they had not engaged in any aerobic or resistance training program in the last 12 months. In this study, we decided to investigate only young men to eliminate the influence of the hormonal variations observed in women due to the menstrual cycle, since female hormones have been associated with different leukocyte responses during exercise-induced muscle injury [56]. Individuals with muscle injury, endocrine disease, and hormonal or nutritional supplement usage were excluded from the study. The participants were randomly divided into two groups: a control group (n = 10) and a group supplemented with fish oil, a natural source of *n-3* PUFAs (n = 11). At the end of the experimental protocol, 2 participants from the control group and 3 from the fish oil group were excluded from the study for different reasons: withdrawal from participating in the study (1 from the fish oil group), an inability to attend the strength-training protocol (at least 85% of participation; 2 from the control group and 1 from the fish oil group), and inadequate supplementation (at least 90% adherence, as assessed by the capsule count at the end of the experimental protocol; 1 from the fish oil group). Thus, at the end, 8 participants of each group completed the experimental protocol, and their results were used in the analysis.

### 2.2. A Single Bout of a Strength Exercise Protocol

A single bout of strength exercises, consisting of 6 sets of 10 maximum repetitions, with intervals of 1 min between sets, was applied at baseline (the pre-training period) and after six weeks of training (the post-training period). The temporal responses (before and 0, 24, and 48 h after the single session) of muscle damage markers—the plasma activity of CK and LDH, and the circulating concentration of inflammatory cytokines (IL-6, TNF-α, and IL-1β) and CRP—were monitored, according to previous studies [5,57]. Plasma cortisol and testosterone levels were measured before and immediately after the single session of strength exercises, and the redox parameters were only measured after 24 h.

### 2.3. Strength-Training Protocol

All participants were supervised by a well-experienced professional in strength training, for the whole training protocol period (6 weeks), which was performed at the Cruzeiro do Sul University (Sao Paulo, Brazil). As an exclusion criterion, a minimum participation of 85% was required for the entire strength-training protocol. All participants were submitted to a strength-training protocol, which comprised a daily undulating periodization model [17] for six weeks, three times per week. Briefly, the participants performed the following training schedule: weeks 1, 3, and 6 (hypertrophy)—6 series of 10 repetitions with a 1 min interval (6 × 10 with 1 min interval); weeks 2 and 4 (strength)—5 × 5 with a 3 min interval; and week 5 (resistance)—2 × 20 with a 1 min interval.

### 2.4. Supplementation with Fish Oil

Fish oil capsules were provided by the Naturalis Nutricao & Farma LTDA (Sao Paulo, Brazil). The participants received 3 capsules of fish oil per day as recommended by the manufacturer. As demonstrated in previous studies, nutritional intervention or fish oil supplementation changes the fatty acid profile after a few weeks [53,58]. A high-performance liquid chromatography (HPLC) analysis for the determination of the fatty acid profile in the fish oil capsules demonstrated that each capsule contained 260 mg EPA and 202 mg DHA. Therefore, the daily doses of *n-3* PUFAs were 780 mg of EPA and 606 mg of DHA. The participants were supplemented for the six weeks of the daily undulating strength training. At the end of the experimental protocol, the remaining fish oil capsules were counted to determine the adherence of the participants to the fish oil supplementation. One participant was excluded because he had less than 90% adherence.

### 2.5. Blood Collection and Plasma Separation

The participants were instructed to not eat for at least four h before blood collection for a biochemical analysis of the plasma. The participants were instructed to have their regular breakfast after waking up (up to 07:00–08:00 a.m.), and blood collection was performed between 11:00 and 12:00 a.m.; therefore, all participants were in the same feeding state. Samples were collected before and 0, 24, and 48 h after a single session of a bout of strength exercise. After that, the blood samples were immediately processed for plasma separation, which was aliquoted and kept at −80 °C until analysis.

### 2.6. Measurements of Plasma Cytokines and C-Reactive Protein

IL-6, TNF-α, and IL-1β were measured using a quantitative immunoassay, an Enzyme-Linked Immunosorbent Assay (ELISA), with kits obtained from R&D System (Minneapolis, MN, USA). The concentration of plasma CRP was determined using a commercial kit from Bioclin (Belo Horizonte, Minas Gerais, Brazil) with immunoturbidimetry.

### 2.7. Plasma Activities of Creatine Kinase and Lactate Dehydrogenase

The activities of plasma CK and LDH were measured using a commercial kit from Bioclin (Belo Horizonte, Minas Gerais, Brazil). CK catalyzes the dephosphorylation of creatine phosphate with the production of adenosine triphosphate (ATP), which reacts with glucose in hexokinase, forming glucose-6-phosphate (G6P). Glucose-6-phosphate dehydrogenase oxidizes G6P to 6-phosphogluconate, reducing nicotinamide adenine dinucleotide (NAD^+^) to NADH, which can be detected via spectrophotometry at 340 nm. LDH catalyzes the pyruvate reduction using NADH, producing lactate and NAD^+^. The decomposition of NADH is proportional to the enzyme activity, and it can be measured at 340 nm.

### 2.8. Measurements of Cortisol and Testosterone

The plasma concentrations of testosterone and cortisol were determined using ELISA, following the specifications of the kits from Cayman Chemical Company (Ann Arbor, MI, USA), according to the manufactures’ instructions.

### 2.9. Determination of Redox State Parameters

#### 2.9.1. Total Iron Determination

The plasma total iron concentration was determined using a kit from Doles-Bioquímica Clínica (Goiania, Brazil). The Fe^2+^:ferrozine complex formed after reducing the ferric ions (Fe^3+^) released from several sources during exercise was measured at 560 nm. The reducing system comprises 0.36 M hydroxylamine chloride, 0.10 M glycine, 14 mM thiosemicarbazide, and 0.50 mM octylphenoxypolyetoxyethanol, at pH 2 [59]. The specific effects of exercise on redox parameters and background levels in rested subjects were normalized to 1.0, and post-exercise values are, thus, expressed as relative values (compared to pre-exercise values). Areas under curves were calculated between background levels (pre-exercise) and 24 h post-exercise levels (AUCpre-24 h).

#### 2.9.2. Heme Iron Determination

Plasma heme iron (from hemoglobin, myoglobin, and other heme proteins) was assayed using a method based on heme iron oxidation by the ferricyanide anion contained in a solution of 0.10 M KH_2_PO_4_, 60 mM K_3_[Fe(CN)_6_], 77 mM KCN, and 82 mM Triton X-100. Heme iron cyanide is stoichiometrically detected at 540 nm, using hemoglobin as a standard curve. The background levels in rested subjects were normalized to 1.0, and post-exercise values are, thus, expressed as relative values (compared to pre-exercise values). Areas under curves were calculated between background levels (pre-exercise) and 24 h post-exercise levels (AUCpre-24 h).

#### 2.9.3. Plasma Trolox Equivalent Antioxidant Capacity (TEAC)

The Trolox equivalent antioxidant capacity in plasma was assayed as described by Van den Berg et al. [60]. Briefly, a 2,2′-azinobis-(3-ethylbenzthiazoline-6-sulfonate radical solution (ABTS^−^) was prepared by mixing 2.5 mM 2,2′-azobis-(2-amidinopropane) and HCl (ABAP) with 20 mM ABTS stock solution in 100 mM phosphate buffer (pH 7.4), containing 150 mM NaCl (PBS). The solution was heated for 12 min at 60 °C, protected from light, and stored at room temperature, and absorbance at 734 nm should be 0.35–0.40 to ensure sufficient ABTS—formation. Since ABTS—gradually decomposes (approximately 2% per hour), regular blanks (in the absence of samples) were recorded for appropriate subtractions. The background levels in rested subjects were normalized to 1.0, and post-exercise values are, thus, expressed as relative values (compared to pre-exercise values). Areas under curves were calculated between background levels (pre-exercise) and 24 h post-exercise levels (AUCpre-24 h).

#### 2.9.4. Reduced and Oxidized Glutathione Measurements

The reduced (GSH) and oxidized (GSSG) glutathione content in plasma was measured as described by Rahman et al. (Rahman et al., 2006) [61]. The method is based on the reaction of reduced thiol groups (such as in GSH) with 5,5′-dithiobis-2-nitrobenzoic acid (DTNB) to form 5-thio-2-nitrobenzoic acid (TNB), which is stoichiometrically detected via absorbance at 412 nm. Purified GSH and GSSG were used as standards. The background levels in rested subjects were normalized to 1.0, and post-exercise values are, thus, expressed as relative values (compared to pre-exercise values). Areas under curves were calculated between background levels (pre-exercise) and 24 h post-exercise levels (AUCpre-24 h).

### 2.10. Statistical Analysis

The results are presented as mean ± standard error of the mean (S. E. M.) and analyzed using Student’s *t*-test when comparing AUC changes (pre- and post-training) between the fish oil and control groups and using two-way ANOVA, followed by Bonferroni post-test for multiple comparisons to evaluate the effect of training and/or supplementation (control pre-training vs. control post-training; fish oil pre-training vs. fish oil post-training; control pre-training vs. fish oil pre-training; and control post-training vs. fish oil post-training). The Cohen’s *d* effect size values were determined based on the mean differences between the fish oil and control groups and pooled SD: Cohen’s *d* = (*M*_2_ − *M*_1_)/*SD*_pooled_; *SD*_pooled_ = √((*SD*_1_^2^ + *SD*_2_^2^)/2) [62,63].

## 3. Results

### 3.1. Plasma Activity of Creatine Kinase and Lactate Dehydrogenase

**Figure 1:**
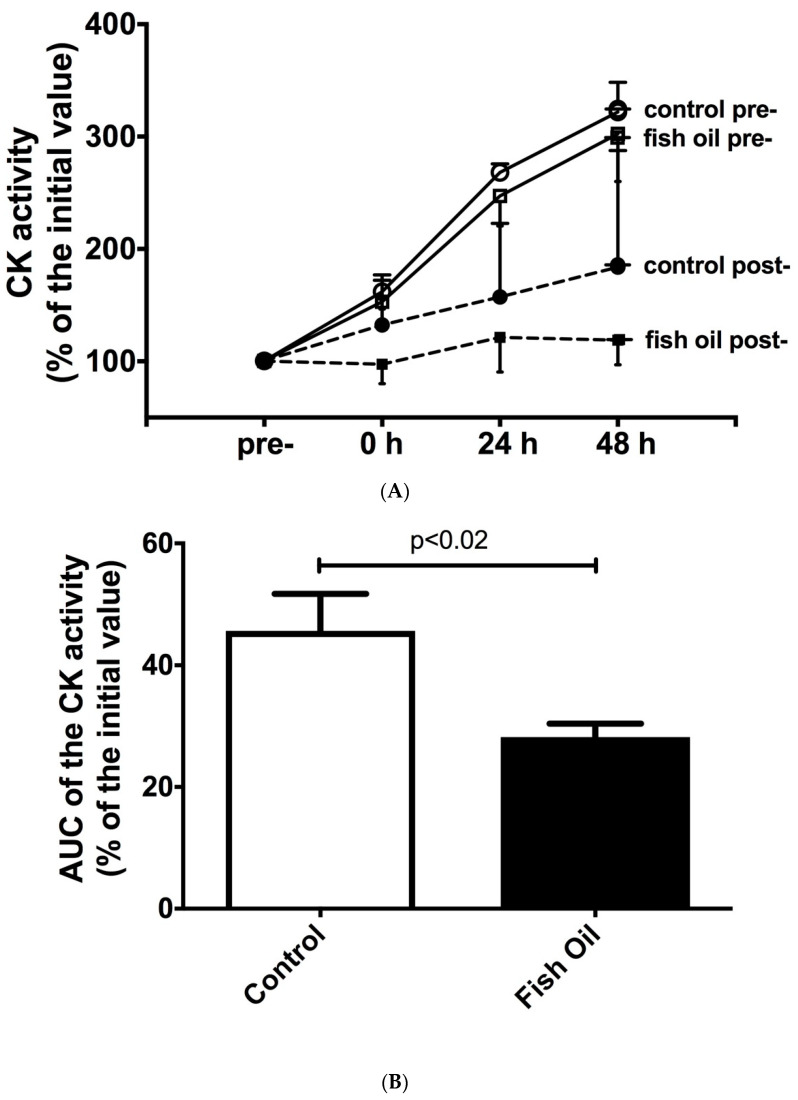

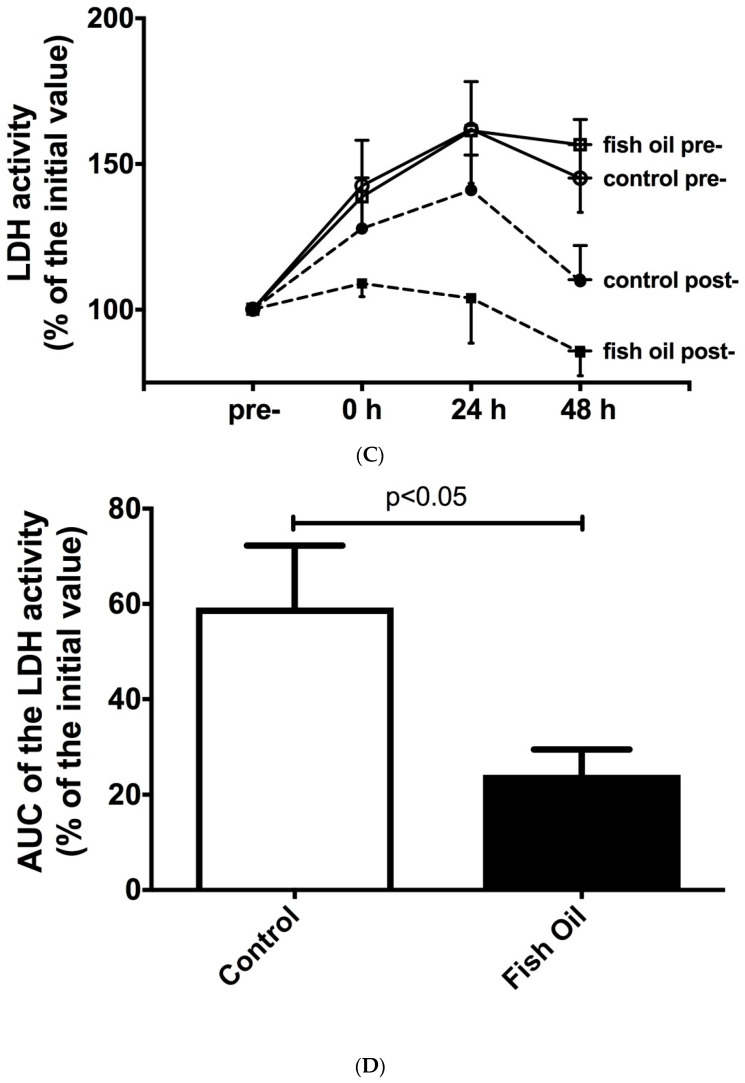
Effect of the fish oil supplementation on plasma activity of (**A**) creatine kinase (CK) and (**C**) lactate dehydrogenase (LDH), in response to a single bout of strength exercises, at baseline (pre-training period) and after 6 weeks of daily undulating strength training (post-training period). On the left, time-dependent plasma CK and LDH activities (before and 0, 24, and 48 h after a single bout of strength exercises). On the right, decrease in the area under curve (AUC) of the temporal plasma CK (**B**) and LDH (**D**) activities after 6 weeks of daily undulating strength training. Results presented as mean ± S.E.M. *p* < 0.02 for CK activity, and *p* < 0.05 for LDH activity, comparing control group with fish oil group.

A single bout of strength exercise increased the CK and LDH activities in the control and *n-3* PUFA-fed groups at baseline (the pre-training period), as shown in Figure 1A,C, respectively. During the post-training period, this increase was attenuated in the control group and reduced by the fish oil supplementation. We did not find any statistical difference using the two-way ANOVA test, but when the AUCs of the control group and the fish oil group were compared using Student’s *t*-test, we observed a marked difference, as demonstrated in Figure 1B,D. The AUCs of the CK and LDH activities were also analyzed using Cohen’s *d* effect size; the supplemented group exhibited a higher attenuation than the control group (effect sizes of −1.44 and −1.40, respectively). The intra-assay coefficient of variance (CV%) was 3.6–7.0% for CK activity and 4.4–9.0% for LDH activity.

### 3.2. Determination of Inflammation Markers

**Figure 2:**
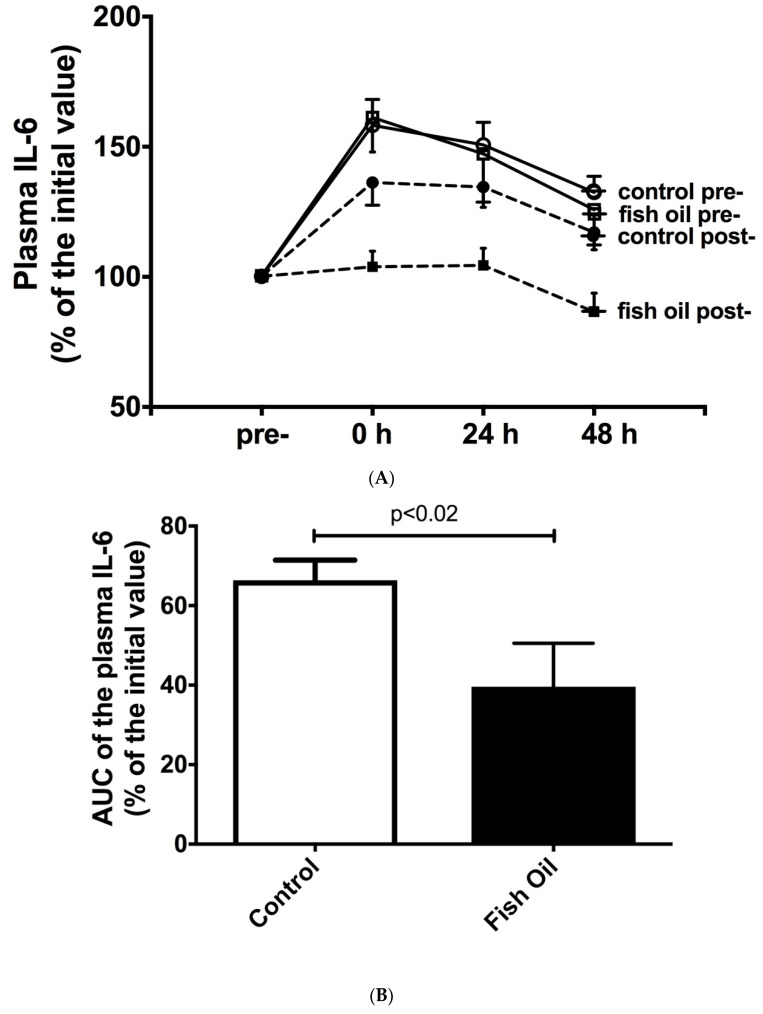

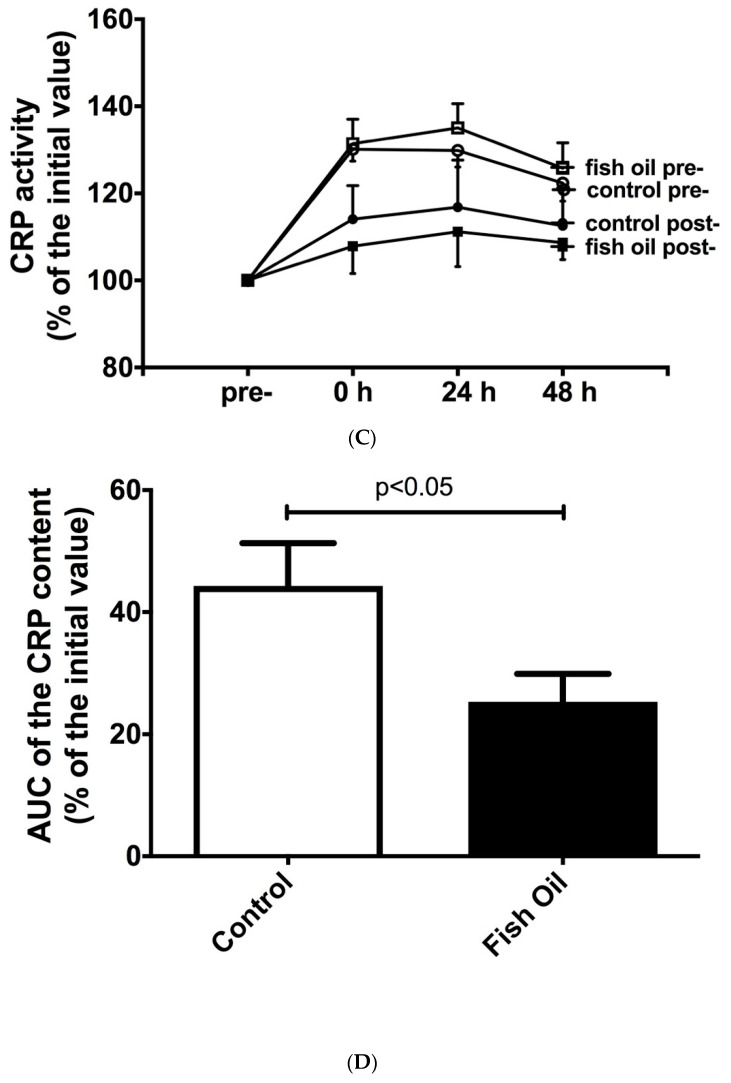
Effect of fish oil supplementation on plasma interlekin-6 (IL-6) (**A**) and C-reactive protein (CRP) (**C**) concentrations, in response to a single bout of strength exercises, at baseline (pre-training period) and after 6 weeks of non-linear strength training (post-training period). On the left, time-dependent plasma concentration (before and 0, 24, and 48 h after a single session of hypertrophic exercises). On the right, reduction in the area under curve (AUC) of the temporal plasma concentrations of IL-6 (**B**) and CRP (**D**) after 6 weeks of daily undulating training, associated or not with fish oil supplementation. Results presented as mean ± S.E.M. *p* < 0.02 for IL-6, and *p* < 0.05 for CRP, comparing control group with fish oil group.

A single bout of strength exercises increased the plasma concentrations of IL-6 and CRP in both groups at baseline (the pre-training period). Following the strength-training protocol (the post-training period), this increase was significantly attenuated in the control group and exacerbated in the fish-oil-supplemented group (Figure 2A,C). No difference was found using the two-way ANOVA test, but a marked reduction was observed when the AUCs of the control group and the fish oil group were compared using Student’s *t*-test, as demonstrated in Figure 2B,D. When the AUCs of the plasma IL-6 and CRP levels were compared using Cohen’s *d* effect size, the supplemented group showed a higher reduction than the control group (effect sizes of −1.30 and −1.21, respectively). The linearity (r^2^) for the IL-6 assay was 0.983. The intra-assay coefficient of variance (CV%) was 4.2–8.50% for IL-6 and 1.1–3.9% for CRP. We did not observe any significant alteration in the plasma IL-1b and TNF-α levels.

### 3.3. Plasma Testosterone: Cortisol Ratio

**Figure 3:**
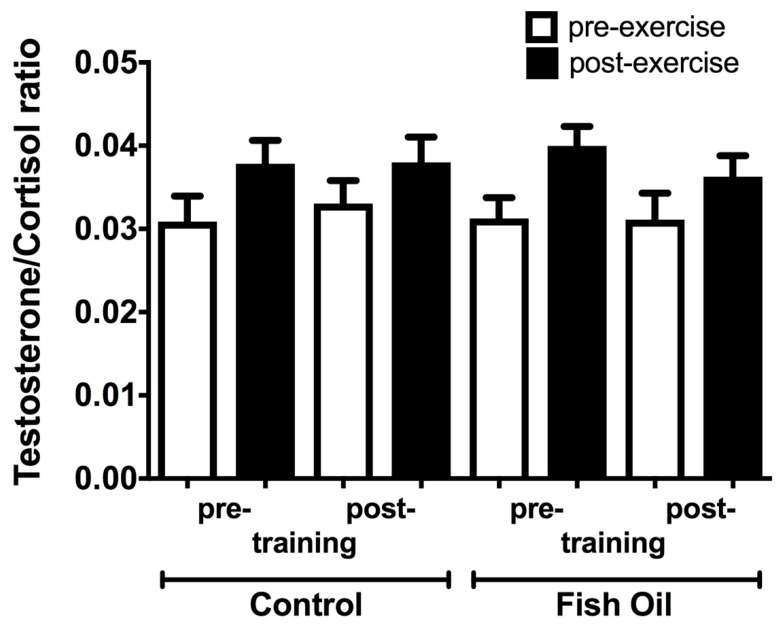
Effect of fish supplementation on plasma testosterone:cortisol ratio in response to a single bout of strength exercise before (pre-training) and after (post-training) 6 weeks of daily undulating strength training. Results presented as mean ± S.E.M.

The testosterone: cortisol ratio was not significantly modified by a single bout of strength exercises before (pre-training) and after (post-training) six weeks of the strength-training protocol. Fish oil supplementation did not alter this response (Figure 3). The linearity (r^2^) for the testosterone assay was 0.991, and for the cortisol assay, it was 0.966. The intra-assay coefficient of variance (CV%) was 4.1–6.2.0% for testosterone and 3.7–8.3% for cortisol.

### 3.4. Measurement of Plasma Redox Parameters

**Figure 4:**
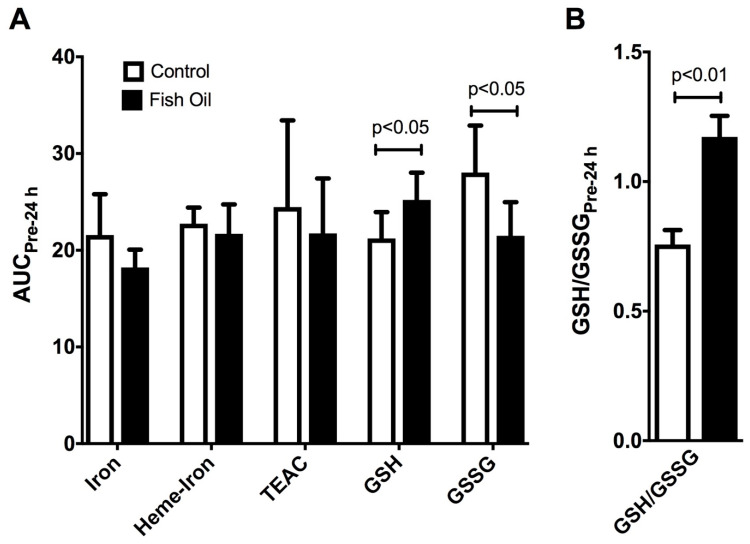
Effect of fish oil supplementation on redox parameters in response to a single bout of strength exercise after 6 weeks of daily undulating strength training (post-training period). Results presented as mean ± S.E.M. (**A**) Areas under curves (AUC) were calculated to express total concentrations of oxidative stress biomarkers in plasma pre- and 24 h post-exercise; (**B**) Reduced/oxidized glutathione ratios (GSH/GSSG) pre- and 24 h post-exercise.

After six weeks of daily undulating strength training, it was found that fish oil supplementation did not modify the plasma concentrations of iron, heme iron, and TEAC after 24 h of a single bout of strength exercises when compared to those of the control group, using Student’s *t*-test. However, it significantly increased GSH and decreased GSSG levels (effect sizes of +1.44 and −1.55, respectively) (Figure 4A). Consequently, the ratio of GSH/GSSH was increased by fish oil supplementation (an effect size of +5.97) (Figure 4B).

## 4. Discussion

Various previous studies have demonstrated the beneficial effects of fish oil supplementation on markers of muscle injury (plasma CK and LDH activities), inflammation (plasma levels of pro-inflammatory cytokines), muscle soreness, and oxidative stress induced by different protocols of a single bout of damaging exercises, both in untrained and trained participants, including athletes [28,50,51,52,53,54]. In these studies, the participants were supplemented with fish oil prior to a protocol of a single bout of exercise that induces muscle damage. The main novelty of our study is that it addresses the effect of fish oil supplementation in combination with strength exercise training on exercise-induced muscle damage in untrained participants. Thus, our study is particularly important because it demonstrates that, in untrained young men submitted to strength exercise training, fish oil supplementation is ideal for alleviating the muscle injury, inflammation, and redox balance induced by a single bout of intense strength exercises.

At baseline (the pre-training period), a single bout of strength exercise increased plasma CK and LDH activities and IL-6 and CRP concentrations, classical markers of muscle damage and inflammation, respectively. However, these effects were significantly attenuated after six weeks of daily undulating training (the post-training period), demonstrating a protective muscle adaptation to the training. The RBE occurs when the individual presents attenuation in muscle injury, inflammation, and soreness after the same or similar bouts of physical exercise or training over time. Thus, the RBE is an important physiological adaptation to protect the skeletal muscle against excessive damage and inflammation, reducing the soreness and muscle recovery time after successive bouts of the same or similar physical exercise sessions or training [16,17,18]. Two components are mainly involved in the RBE in our study: (i) the first bout of strength exercise and (ii) the strength training. Regular physical training promotes anti-inflammatory and antioxidant responses [64,65], which additionally contribute to the RBE.

After muscle damage, an adequate and well-controlled inflammatory response is required to completely restore muscle homeostasis and for recovery [1]. This response involves the recruitment of leukocytes into injured tissue and the production of pro-inflammatory cytokines, consequently increasing these mediators’ local and systemic concentrations [1,10]. However, an exacerbated inflammatory response after eccentric exercises can impair or delay muscle repair and regeneration. In our study, we observed an increased temporal plasma release of IL-6 and CRP after a single bout of strength exercises, but there were no differences in TNF-α or IL-1β plasma concentrations. Previous studies have also found no alterations in pro-inflammatory cytokines induced by physical activity [5,14]. The pro-inflammatory cytokines TNF-α, IL-1β, and IL-6, are essential for the acute inflammatory response, as they stimulate the production of acute-phase proteins, including CRP. This response depends on the characteristics of the physical exercise involved, including the intensity, volume, and intervals among series [15,66]. Other authors suggest that pro-inflammatory cytokines are locally produced by the exercised muscles and released into circulation but rapidly degrade, remaining stable in plasma for a short period [8]. These observations can explain, at least in part, our results concerning the pro-inflammatory cytokines IL-1β and TNF-α.

Supplementation with *n-3* PUFA additionally increased the RBE, as demonstrated by the reduction in the plasma activities of CK and LDH and the circulating concentrations of IL-6 and CRP. Although the mechanisms involved in the RBE are not entirely known yet, cellular modifications may occur as a result of the fish oil supplementation, improving the protective adaptation against muscle damage induced by strength exercises. Some studies suggest that the increased recruitment of sarcomeres during contraction decreases mechanical stress, avoiding the rupture of myofibrils [67]. The reduced inflammatory process in the participants submitted to the fish oil supplementation could further attenuate the response induced by strength exercises.

The relationship between testosterone and cortisol in response to physical exercise indicates physical stress or an imbalance between anabolic and catabolic processes [68,69]. We did not observe any alteration in the testosterone/cortisol ratio as a result of the strength-training protocol or the fish oil supplementation, suggesting that our experimental protocol could not modify physical stress or the anabolic/catabolic balance. Uchida et al. [57] evaluated the influence of different intensities (50, 75, 90, and 110% of 1RM) of the bench press exercise on the same hormones, and they also did not find any changes in the plasma concentrations of both steroid hormones. The authors suggested that the possible cause of this effect was the low volume of exercise and muscle mass involved in the bench press exercise. Crewther et al. [70] studied the impact of three different sessions of squat exercises (45%, 75%, and 88% of 1RM) on plasma testosterone and cortisol concentrations. Interestingly, the session of 75% led to the highest increase compared to the other sessions. Thus, the modulation of the testosterone/cortisol ratio depends on the experimental protocol.

Several authors have used antioxidant compounds to reduce oxidative stress induced by physical exercise [71,72], including interventions with fish oil [25,26,27,28]. Regarding redox parameters at the end of the non-linear strength training in our study, no changes in the plasma concentrations of iron, heme iron, and TEAC were observed as a result of the fish oil supplementation. However, increased GSH, decreased GSSG, and consequently an increased GSH/GSSG ratio were found in the supplemented group, suggesting an improved antioxidant defense. GSH rapidly reacts nonenzymatically with reactive oxygen/nitrogen species (ROS/RNS), including the hydroxyl radical, dinitrogen trioxide (N2O3), and peroxynitrite [73]. Moreover, GSH also participates in enzymatic antioxidant defense, e.g., as a substrate of the GPx-mediated reduction of peroxides, resulting in the production of GSSG. The fish oil supplementation improved the GSH/GSSG antioxidant system. An elevated GSH/GSSG ratio is required to control the reducing environment [74]. The effects of *n-3* PUFAs and/or physical exercise might be effective under conditions of an impaired redox balance [26], including in older people [75] and in metabolic and inflammatory diseases [47,76].

**Figure 5:**
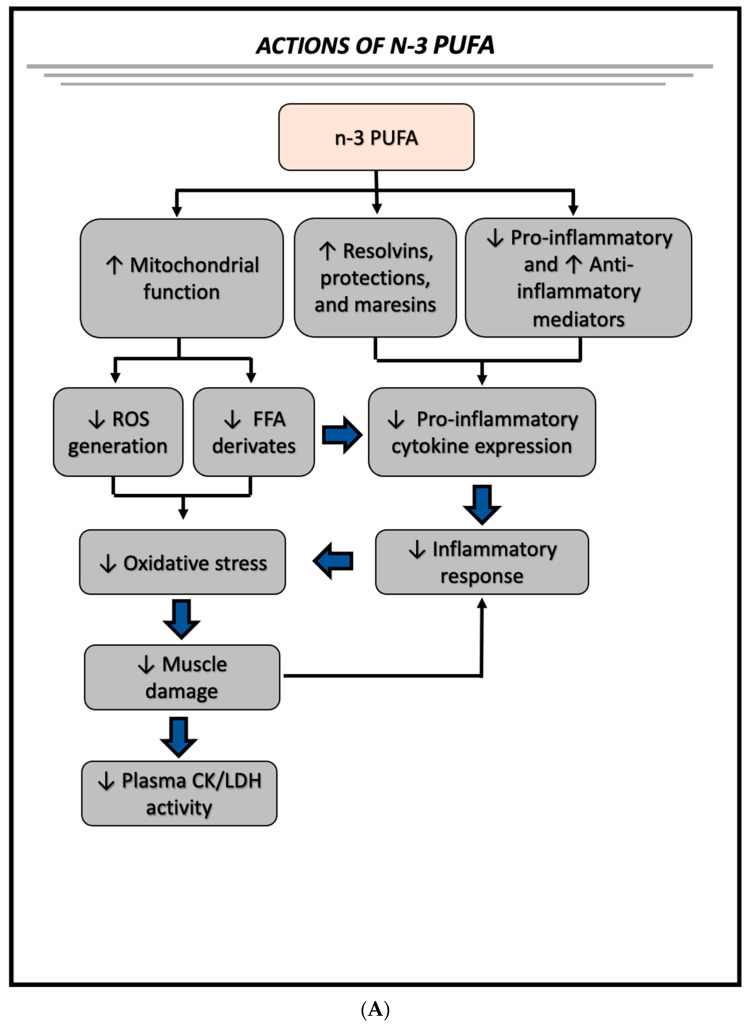

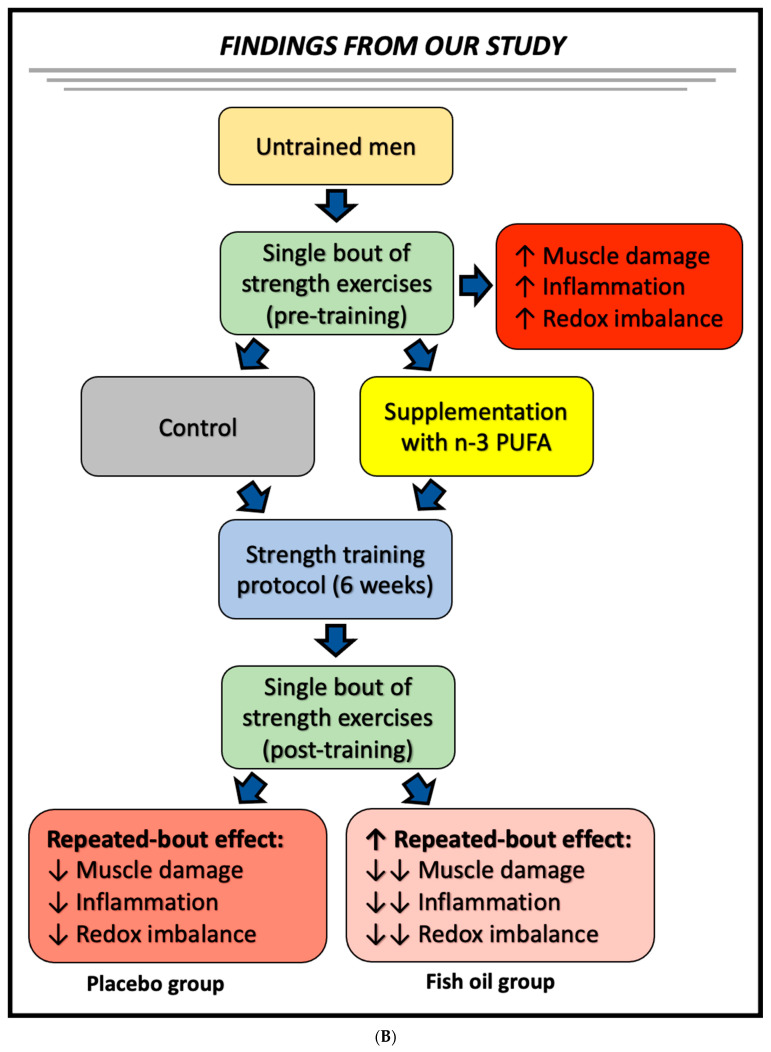
Actions of *n-3* PUFA (**A**) and effects of *n-3* PUFA supplementation on muscle damage induced by a single session of strength exercises after 6 weeks of non-linear strength training (post-training period) (**B**).

The anti-inflammatory effect of *n-3* PUFA has been demonstrated by various research groups, and it has been related to the beneficial effects of these metabolites in different inflammatory diseases, obesity, diabetes mellitus, metabolic syndrome, cardiovascular diseases, fatty liver disease, and cancer [77,78,79,80]. The mechanisms of action of *n-3* PUFA involve several signaling pathways, including the activation of GPR120 [81], the generation of anti-inflammatory and/or pro-resolution lipid mediators (resolvins, protectins, and maresins) [82], and the reduction of pro-inflammatory lipid derivatives (prostaglandin and thromboxane 2 series, and leukotriene 4 series) [83]. Our group also demonstrated that *n-3* PUFA supplementation improves mitochondrial function in the skeletal muscle of an animal model of high-fat diet-induced obesity [34]. We propose herein that *n-3* PUFA could potentialize the repeated-bout effect induced by strength training through several actions, including (1) the anti-inflammatory effect, reducing the production of pro-inflammatory cytokines; (2) improved mitochondrial function in skeletal muscle, decreasing the generation of lipid derivatives and reactive oxygen species; and (3) decreased oxidative stress, resulting in diminished muscle damage. This proposition and the main findings of this work are summarized in Figure 5.

Our study is the first to demonstrate the beneficial effects of fish oil supplementation in combination with a strength-training protocol for 6 weeks on the muscle damage markers, inflammation, and redox imbalance induced by a single bout of strength exercises. It is important to describe some of the limitations of our study. First, we investigated the effects of *n-3* PUFAs and strength training for a short period (6 weeks); further studies are required to evaluate the effects for longer periods. Second, we assessed the adherence to the fish oil supplementation only by counting the remaining fish oil capsules at the end of the experimental protocol; a direct measurement (e.g., the determination of plasma fatty acid profiles) is lacking. Third, we analyzed only young men; further studies are required to analyze young women at different phases of the menstrual cycle and other groups of participants, including older people. Lastly, we used a small sample size (n = 8 per group), which could have reduced the statistical power of our analysis. However, the Cohen’s *d* effect size values of our data (*d* > 1.0) suggest a large effect of the fish oil supplementation. In addition, the effects of fish oil supplementation on exercise-induced muscle injury were also observed in previous studies that used a similar number of participants (n = 7–11 per group) to demonstrate the effects of the supplementation [53,54,55]. Thus, based on the findings of previous studies, the well-controlled strength-training protocol that we used, and the Cohen’s *d* effect size values that we found, our results seem to be statistically representative.

In summary, supplementation with *n-3* PUFAs improved the RBE and redox parameters in healthy young men submitted to daily undulating training for six weeks, as demonstrated by the decreased muscle damage (plasma activities of CK and LDH), pro-inflammatory markers (IL-6 and CRP), and redox biomarkers (increased GSH/GSSG ratio) after a bout of strength exercises. Thus, our study is of particular interest because it demonstrates that, in untrained young men submitted to a strength-training protocol, fish oil supplementation is ideal for alleviating the muscle injury, inflammation, and redox imbalance induced by a single session of intense strength exercise. Our findings highlight fish oil supplementation as an effective nutritional strategy to reduce the muscle damage, inflammation, and redox imbalance in untrained individuals who intend to engage in strength-training programs. Further studies are necessary to determine the persistence of this modulation for prolonged training periods and the effects of fish oil supplementation combined with strength exercise training in other groups of participants, including young women and older people.

## Data Availability

The data presented in this study are available in the article.
